# An Improved Chloroplast DNA Extraction Procedure for Whole Plastid Genome Sequencing

**DOI:** 10.1371/journal.pone.0031468

**Published:** 2012-02-22

**Authors:** Chao Shi, Na Hu, Hui Huang, Ju Gao, You-Jie Zhao, Li-Zhi Gao

**Affiliations:** 1 Plant Germplasm and Genomics Center, Kunming Institute of Botany, Chinese Academy of Sciences, Kunming, China; 2 Graduate School of Chinese Academy of Sciences, Beijing, China; University of Georgia, United States of America

## Abstract

**Background:**

Chloroplast genomes supply valuable genetic information for evolutionary and functional studies in plants. The past five years have witnessed a dramatic increase in the number of completely sequenced chloroplast genomes with the application of second-generation sequencing technology in plastid genome sequencing projects. However, cost-effective high-throughput chloroplast DNA (cpDNA) extraction becomes a major bottleneck restricting the application, as conventional methods are difficult to make a balance between the quality and yield of cpDNAs.

**Methodology/Principal Findings:**

We first tested two traditional methods to isolate cpDNA from the three species, *Oryza brachyantha*, *Leersia japonica* and *Prinsepia utihis*. Both of them failed to obtain properly defined cpDNA bands. However, we developed a simple but efficient method based on sucrose gradients and found that the modified protocol worked efficiently to isolate the cpDNA from the same three plant species. We sequenced the isolated DNA samples with Illumina (Solexa) sequencing technology to test cpDNA purity according to aligning sequence reads to the reference chloroplast genomes, showing that the reference genome was properly covered. We show that 40–50% cpDNA purity is achieved with our method.

**Conclusion:**

Here we provide an improved method used to isolate cpDNA from angiosperms. The Illumina sequencing results suggest that the isolated cpDNA has reached enough yield and sufficient purity to perform subsequent genome assembly. The cpDNA isolation protocol thus will be widely applicable to the plant chloroplast genome sequencing projects.

## Introduction

Chloroplasts (plastids) are plant organelles that contain a circular DNA containing ∼130 genes with the size ranging from 72 to 217 kb [1], [2]. cpDNAs of green plants are exceptionally conserved in their gene content and organization, providing sufficient information for genome-wide evolutionary studies. Recent efforts have proven their potentials in resolving phylogenetic relationships at different taxonomic levels and understanding structural and functional evolution by using the whole chloroplast genome sequences [3], [4], [5].

Plant cpDNAs have been set as targets among the very early genome sequencing projects owing to their small sizes [6]. To date, at least 200 plant complete cpDNAs have been sequenced (http://www.ncbi.nlm.nih.gov/genomes/GenomesGroup.cgi?taxid=2759&opt=plastid), and in the recent years, the number is rapidly increasing due to an extensive application of the second-generation sequencing technologies to the whole chloroplast genome sequencing. Despite its short sequence reads, excess sequence data produced by the second-generation sequencing technologies are fairly suitable for the genome assembly, because the chloroplast genome is much smaller in size and simple in structural complexity compared to nuclear genomes [7]. For example, a single 600 Gbp per run in the Illumina HiSeq-2000 (http://www.illumina.com) could conceivably sequence ∼40,000 average-sized chloroplast genomes to a depth of 120×. Next-generation sequencing technologies have undoubtedly made it possible to sequence the entire plant genomes more efficiently and economically than ever before with decreased time and costs compared with traditional approaches [6]. With rapid progress in sequencing technologies, the acquisition of high quality cpDNAs from plant tissues for the whole genome sequencing is urgently needed.

Two experimental methods are often employed to collect cpDNAs in plants. The first is the whole chloroplast genome amplification from total DNA using long polymerase chain reaction (PCR), and the second is direct isolation of cpDNAs from fresh plant materials based on sucrose gradient. The former method is PCR-based cpDNA sequencing, which is usually used to the situation that substantial plant leaf materials (e.g., ∼20 to 100 g fresh leaves) are unavailable but can be substituted by extracting total DNA from limited materials. The cpDNA fragments are further amplified by using the conservative primer pairs [8]. The latter focuses on isolating the chloroplasts from fresh plant leaves according to sucrose gradient centrifugation, followed by a direct extraction of cpDNAs from intact chloroplasts [9]. Of them, sucrose gradient centrifugation is limited by the availability of ultracentrifuges which are not facilitated in many laboratories [10]. As a result, the PCR method is the most extensively used among the chloroplasts sequencing projects regardless of its time-consumption and higher costs [11]. As an alternative of the sucrose gradient centrifugation process, DNAse I treatment [12] and high salt precipitation [13] have succeeded in isolating cpDNAs of some specific plant species, but further applications to additional species have been restricted, as both of them were not easy to make a balance between enough yield and quality with limited contamination of nuclear and mitochondria DNA [14].

The rapid progress in the next-generation technologies requires developing new methods to isolate cpDNAs with increased quality and yield, especially aiming to simplify the isolation process so as to meet the need for the whole chloroplast genome sequencing. We modified the above-described methods [9], [12], [13] to develop a new protocol and further applied it to isolate cpDNAs from the three species, *Oryza brachyantha*, *Leersia japonica* and *Prinsepia utihis*. To test their purities, these three isolated cpDNAs were subsequently sequenced by using the Illumina (Solexa) sequencing-by-synthesis technology.

## Results and Discussion

### The isolation of cpDNA

**Figure 1:**
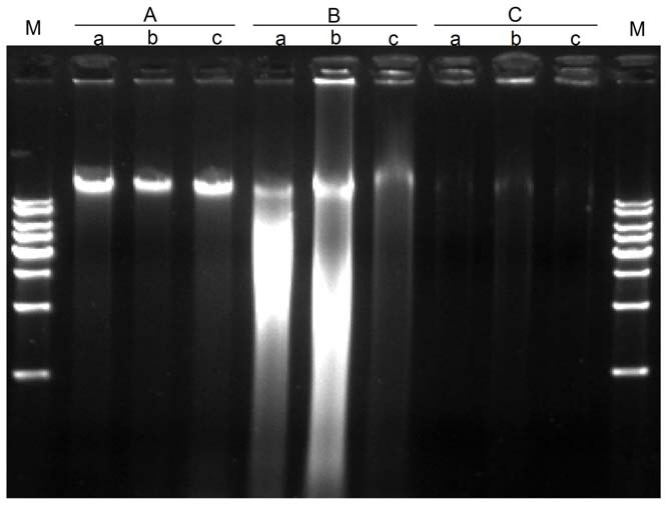
Comparison of cpDNA isolation from the three plant species among different extraction methods. Three methods, including A) modified protocol, B) DNAse I treatment and C) sucrose gradient centrifugation, were separately employed to isolate cpDNAs from a) *O. brachyantha*, b) *L. japonica*, and c) *P. utihis*. For each plant species, 20 g fresh leaves were used. The DNA bands were shown on a 0.8% agarose gel. M indicates 1 kbp DNA ladder.

The cpDNA isolation includes the three basic steps: separation of plastids from leaf tissues, lysis of the chloroplasts, and purification of DNA. Because the isolation of intact chloroplasts often acts as a critical stage of the whole procedure, the method based on sucrose gradient ultracentrifugation is the most commonly employed to effectively separate nuclear DNAs from cpDNAs. Using two grass species (*O. brachyantha* and *L. japonica*) and one rosid plant (*P. utihis*), we first performed cpDNA isolation by following the previously described procedure [9]. Electrophoresis of the resulting DNA displayed a very weak band on agarose gel, indicative of very low cpDNA yield (Figure 1C). This study only used 20 g fresh leaves, while more than 100 g of leaf tissues were recommended [9]. Another possible explanation was that, after the sucrose gradient centrifugation, only a small amount of chloroplast pellet was collected, leading to the extraction of few cpDNAs. Because the library preparation for the whole genome sequencing needs a substantial amount of starting DNA, either repeated cpDNA isolation or substantial leaves are required to use this method. Considering the amount of time-consumed by sucrose gradient preparations, two alternative methods, DNAse I treatment and high salt method, may be suitable to replace the sucrose gradient centrifugation method.

The DNAse I treatment method used DNAse I to digest nuclear DNA that adheres to the outer chloroplast membrane. The success in isolating the cpDNAs was reported from two of many species which they have attempted [9]. When the three plant species were used in this study, however, we failed to isolate intact cpDNAs since they all were degraded by the DNAse I (Figure 1B). The result is consistent with the fact that DNAse I digest not only the nuclear DNA but also the cpDNA which might not be well protected within intact plastids [9], [15].

The second alternative method employs a high NaCl concentration in the isolation buffers, which do not involve any sucrose gradient centrifugation. This method was only reported to have succeeded in isolating the pea cpDNA [13]. Considering that only increasing the NaCl concentration may not be enough to enhance cpDNA purity, we made several modifications of the method to broaden its application to as many taxa as possible. The final protocol (see materials and methods) demonstrated the advantage of isolating sufficient cpDNAs with leaf materials of the same three plant species (Figure 1A).

**Figure 2:**
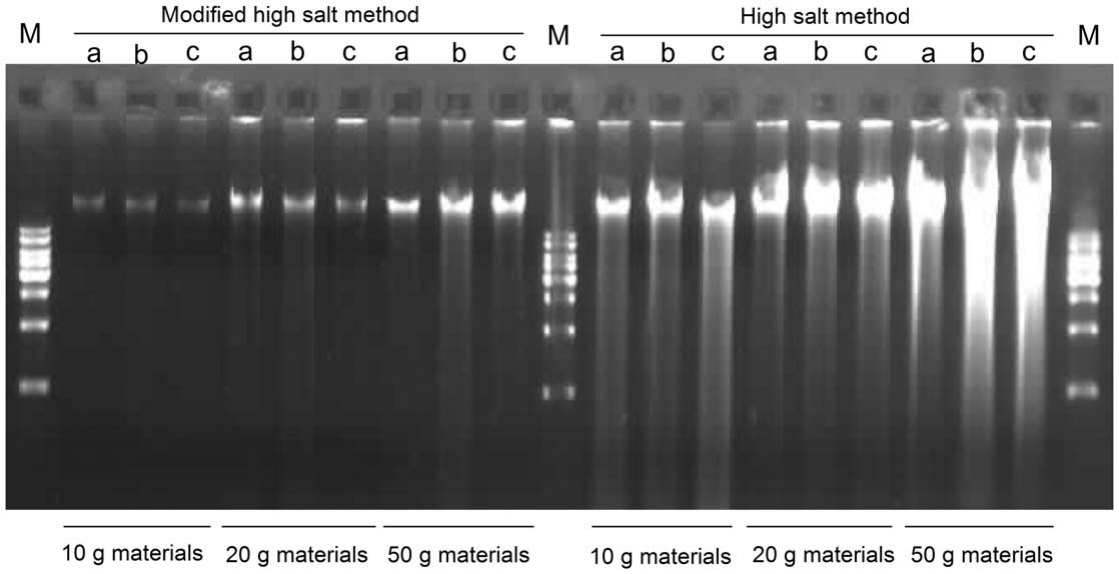
Comparison of cpDNA isolation with high salt method and the modified high salt method for the three plant species. cpDNAs were isolated from 10 g, 20 g, and 50 g fresh leaves of the three plant species: a) *O. brachyantha*, b) *L. japonica*, and c) *P. utihis*. The DNA bands were shown on a 0.8% agarose gel. M indicates 1 kbp DNA ladder.

**Figure 3:**
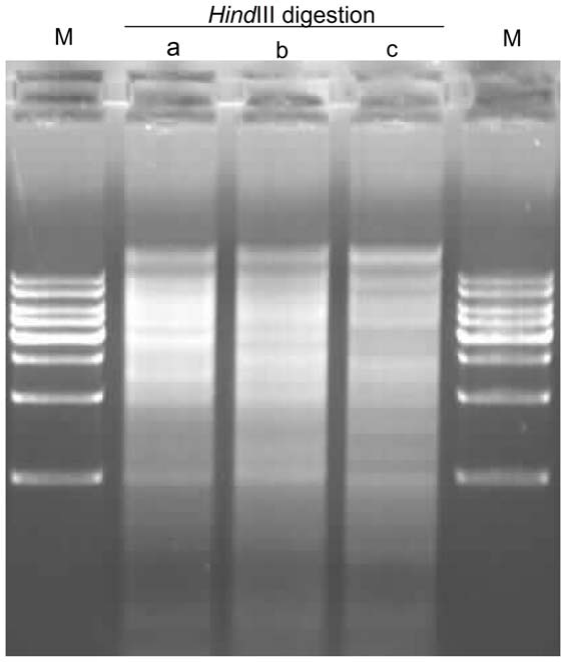
*Hind*III restriction enzyme digestion of isolated cpDNAs from the three plant species. a) *O. brachyantha*, b) *L. japonica*, and c) *P. utihis*. The DNA bands were shown on a 0.8% agarose gel, and DNA was isolated with the improved high salt method; M indicates 1 kbp DNA ladder.

As a modification of the sucrose gradient centrifugation, the high salt method significantly simplified the cpDNA isolation process. By using this method, our first effort to isolate the cpDNAs also seems successful, as it can get a relatively clearly defined DNA band. When we increased the amount of fresh leaves, however, a positive correlation between increased DNA yield and the possibility of DNA degradation was found, indicating that there is more contamination of nuclear DNAs (Figure 2). The observation suggests that this method may not be suitable to isolate cpDNAs with high purity. As an alternative approach in chloroplast isolation, four to six volumes (v/w) cold isolation buffer (in the original protocol) may not be enough to homogenate the fresh leaves (e.g., 20 g fresh leaves with 100 ml isolation buffer). Therefore, we increased the amount of isolation buffer from 5 to 20 volumes of fresh leaves (e.g., 20 g fresh leaves with 400 ml buffer A in our protocol) in the subsequent experiment. Even when 50 g fresh leaves were used, a well-defined cpDNA band can be observed (Figure 2), suggesting that the modification led to a successful isolation of the cpDNAs. It is likely that about 20 g leaves may be more optimal as it could include less contaminating nuclear DNA. Furthermore, two additional centrifugation steps (200 g 20 min and 3500 g 20 min, separately) were used to discard the cell debris and collect chloroplast pellet. To decrease the nuclear DNA contamination that adheres to the outer chloroplast membrane, we also incorporated extra steps to wash the chloroplast pellet with buffer B, further increasing the purity of isolated cpDNAs. Last but not least, chloroplasts were lysed using SDS and Proteinase K instead of cetyltrimethylammonium bromide (CTAB) followed by phenol/chloroform extraction. The final isolated cpDNAs were digested with *Hind*III and the result was visualized on a 0.8% agarose gel (Figure 3). Among these modifications, incorporated gradual centrifugation steps were of the most importance, because they are able to increase the cpDNA purity by separating the chloroplasts from cell debris. If larger amounts of starting materials (e.g., 50 g fresh leaves) were used, it is necessary to add a second centrifugation step at 200 g.

Of these four methods, our modified high salt method was more efficient to isolate the cpDNAs, and most importantly, to balance cpDNA yield and purity to the greatest extent. Indeed, our lab has been employing this improved protocol and extracted hundreds of plant species, which has proved to be highly efficient to isolate cpDNA from more taxa of plants (unpublished data).

### Sequencing chloroplast DNAs using the second-generation illumina sequencing technology

**Figure 4:**
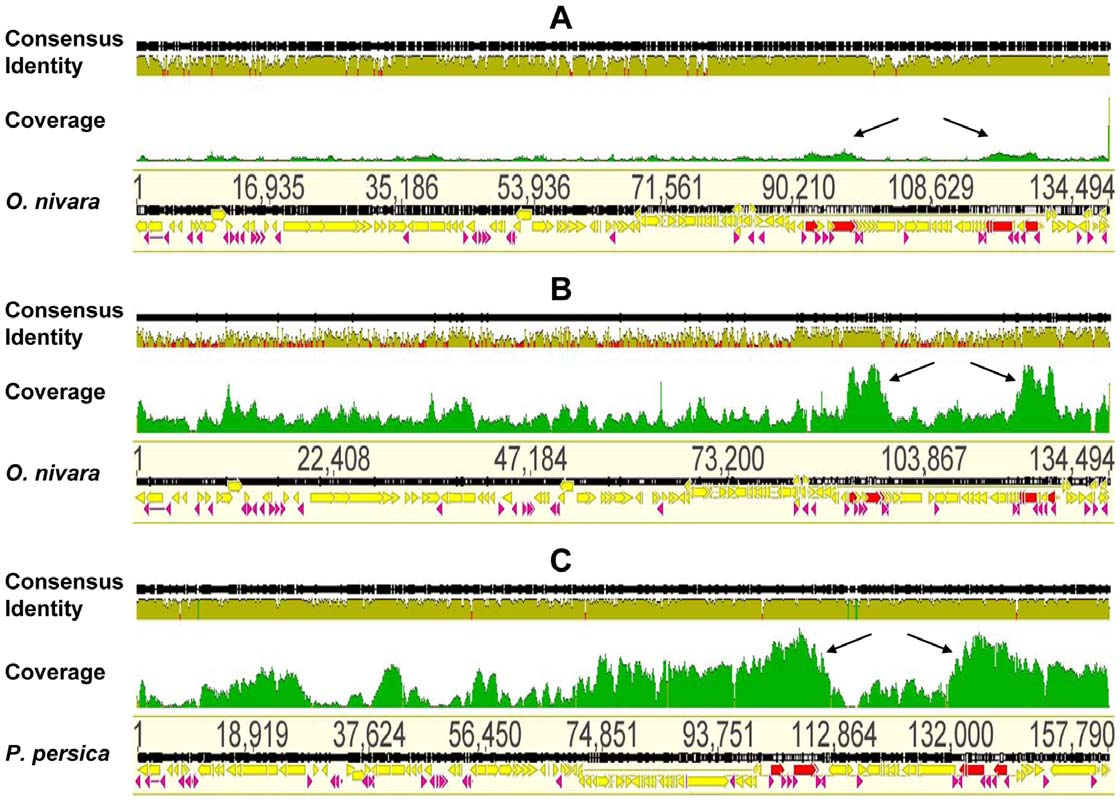
Reference guided chloroplast genome assembly. A) *O. brachyantha* (consensus) sequence reads were aligned to *O. nivara*; B) *L. japonica* (consensus) sequence reads were aligned to *O. nivara*; and C) *P. utihis* (consensus) sequence reads were aligned to *Prunus persica*. The genome coverage is shown as green peaks and arrows indicate regions of high coverage.

The vast improvements made in DNA sequencing technologies offer unprecedented opportunities to perform phylogenomic studies based on the whole chloroplast genome sequences. Multiplex sequencing with the second-generation technology allows multiple samples to be sequenced in a run, generating millions of reads that significantly increase the sequence depth [16]. To test the cpDNA purity isolated by our protocol, in this study, we sequenced these three chloroplast genomes by using Illumina sequencing technology. Sequencing reactions generated a total of 330 Mbp sequence data with 5 Mbp in *O. brachyantha*, 21 Mbp in *L. japonica* and 304 Mbp in *P. utihis* (table 1). A reference-guided chloroplast genome assembly was performed to roughly estimate the genome coverage (figure 4), the *O. brachyantha* (Figure 4A) and *L. japonica* (Figure 4B) were assembled to *O. nivara*, while *P. utihis* (Figure 4C) was assembled to *Prunus persica*.

**Table 1:** Summary of total sequenced data and aligned reads of three plant species.

Species	Total bases (bp)	Total reads	Aligned reads	Aligned (%)	Average coverage	Reference genome
***Oryza brachyantha***	4 956 842	51 606	26 659	51.6	19.0	*O. nivara* NC_005973
***Leersia japonica***	21 321 958	221 544	95 268	43.0	69.2	*O. nivara* NC_005973
***Prinsepia utihis***	303 846 384	3 132 702	1 385 592	44.2	855.7	*P. persica* NC_014697

We surprisingly found that the cpDNA purity, represented by the percentage of the reads aligned to the reference genome, were relatively consistent across the three species, although the amount of sequence data varied greatly among them, ranging from 51, 606 reads in *O. brachyantha* to 3, 132, 702 reads in *P. utihis*. The cpDNA reads were 51.6% in *O. brachyantha*, 43.0% in *L. japonica*, and 44.2% in *P. utihis*, respectively (table 1). The average coverage was only 19× in *O. brachyantha*, as only 5 Mbp were obtained. In *P. utihis*, however, the generation of 304 Mbp led to cpDNA genome coverage of 855.7× (table 1). In this study, all of the reference genomes were sufficiently covered, showing two peaks in the invert repeat (IR) regions (figure 4). Despite the relatively fewer sequences and thus lower genome coverage in *O. brachyantha*, there were no large gaps found in the consensus sequence (Figure 4A). Our results thus suggest that, given the cpDNA purity isolated with this modified method, obtaining 50 Mbp of sequence data could lead to at least 100× average coverage of the chloroplast genome which is sufficient for the assembly.

Previous studies [17], [18] suggested that no more than 5% of cpDNAs usually exists among the total DNA in angiosperms. However, our protocol can efficiently isolate the cpDNAs with percentages of about 40–50% (table 1). The RCA-based (rolling circle amplification) cpDNA sequencing method [9] reported that approximately 10–40% of the resulting RCA products consisted of non-cpDNA [19]. In comparison, our method apparently showed its power in isolating cpDNAs with improved quality and lowered sequencing costs, although there is room to further improve the cpDNA purity.

In conclusion, this study provides a quick and efficient method for isolating cpDNAs from angiosperms. In comparisons with the commonly used methods of sucrose gradient centrifugation and the DNAse I treatment, our modified method indeed works competently when testing with leaf materials of the same three plant species of *O. brachyantha*, *L. japonica* and *P. utihis*. The cpDNA bands could be clearly defined on the agarose gel. By means of the next-generation Illumina sequencing technology, the three isolated cpDNA samples were subsequently sequenced and their purity reached ∼40–50%, which were sufficiently pure to further perform the genome assembly. In addition, we tested the genome coverages influenced by the sequence data, showing that only ∼50 Mbp could attain at least 100× average coverage of the chloroplast genome when the cpDNA purity reached ∼40–50%. In all, this modified method is able to serve as an efficient cpDNA extract procedure to complete the chloroplast genome sequencing of angiosperms.

## Materials and Methods

### Plant materials

The *O. brachyantha* and *L. japonica* (Poaceae) plants were grown in the greenhouse, while *P. utihis* (Rosaceae) was transplanted in Botanical Garden of Kunming Institute of Botany, Chinese Academy of Sciences. For each plant species, ∼20 g of the fresh leaves were collected and cleaned with distilled water, and then they were restored in 4°C refrigerator for further experimental uses.

### Protocols

**Figure 5:**
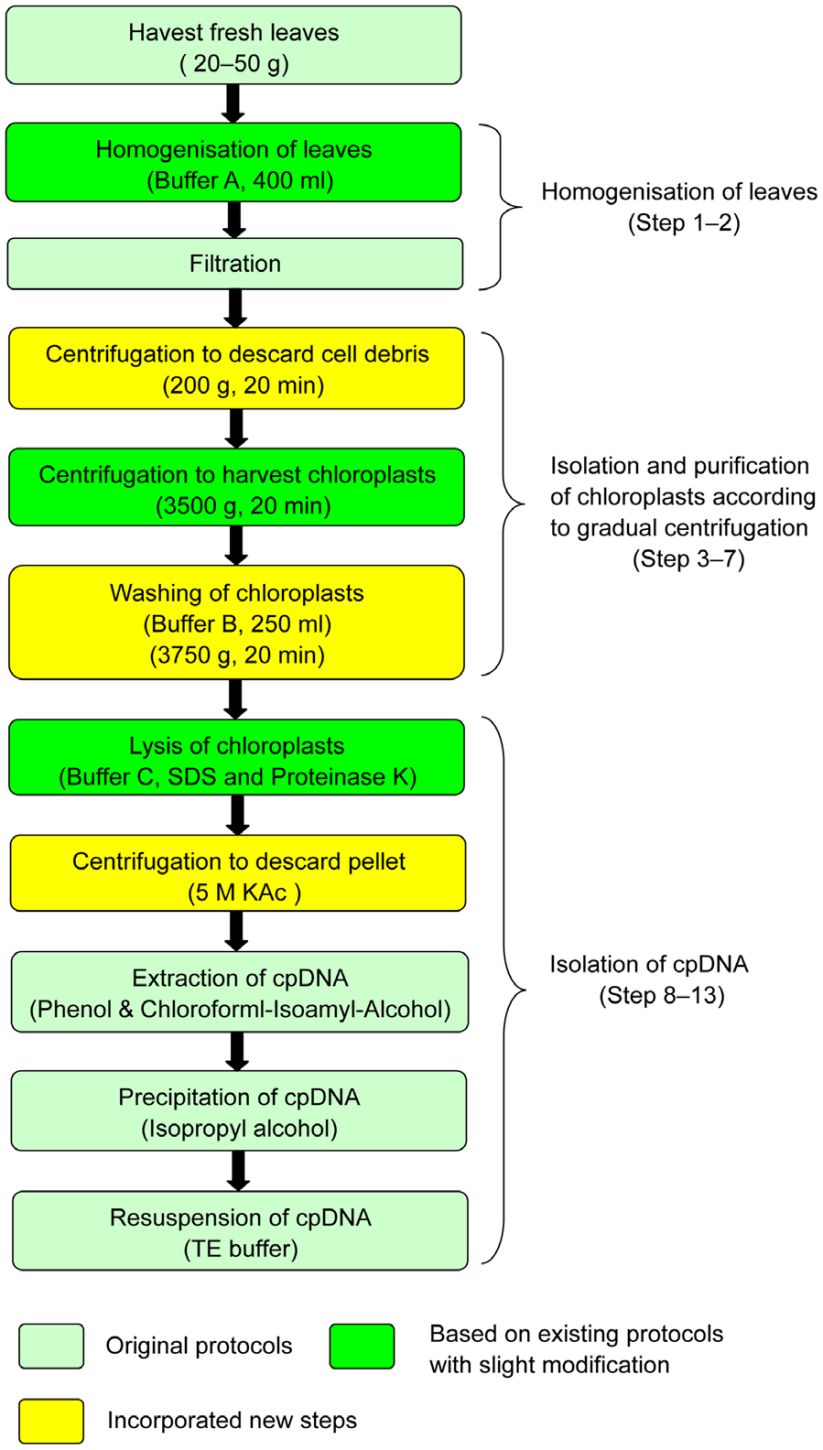
Flowchart showing the major steps for the isolation of cpDNAs using the modified high salt method.

The four cpDNA isolation methods used in our study were described as below:

A. Modified high salt method (Figure 5)

Reagents

Buffer A (PH 3.8)

1.25 M NaCl, 0.25 M ascorbic acid, 10 mM sodium metabisulfite, 0.0125 M Borax, 50 mM Tris-HCl (pH 8.0), 7 mM EDTA, 1% PVP-40 (w/v), 0.1% BSA (w/v), 1 mM DTT;

Buffer B (PH 8.0)

1.25 M NaCl, 0.0125 M Borax, 1% PVP-40 (w/v), 50 mM Tris-HCl (PH 8.0), 25 mM EDTA, 0.1% BSA (w/v), 1 mM DTT;

Buffer C

100 mM NaCl, 100 mM Tris-HCl (PH 8.0), 50 mM EDTA, 1 mM DTT;

Both BSA and DTT were added just before the start of the experiment.

Chloroplast isolation

All the following steps were carried out at 0°C if not otherwise stated.

1. Prior to extraction, about 20 g (fresh weight) leaves were collected and kept in dark for 48 to 72 hours at 4°C to decrease starch level stored in the leaves.

2. The leaves were nervure-removed, cut into pieces (∼1 cm) and homogenized in 400 ml ice-cold buffer A for 30 seconds. Filter the homogenate into centrifuge bottles using two layers of Miracloth (Merck) by softly squeezing the cloth.

3. Centrifuged the homogenate (200 g, 20 min). The nucleus pellet and cell-wall debris were discarded.

4. Repeated the centrifugation once again. The supernatant included chloroplasts suspended in it.

5. Centrifuged the supernatant at a higher centrifugal force of 3500 g for 20 min, the resulting pellet were chloroplast pellet with some contamination of nuclear DNAs.

6. Added 250 ml Buffer B to the pellet and suspend it gently using a paintbrush to wash the nuclear DNAs attaching to the chloroplast cytomembrane. Then centrifuge with 3500 g for 20 min and discard the supernatant.

7. Re-suspended the pellet with 250 ml Buffer B again and centrifuged (3750 g for 20 min) to gain the purified chloroplasts.

Chloroplast DNA isolation

8. Added 8 ml Buffer C, 1.5 ml 20% SDS, 20 µl β-Me, 30 µl Proteinase K (10 mg/ml) to the purified chloroplast pellet and incubate at 55°C for at least 4 hours or overnight. The chloroplasts would be fully lysed.

9. Put the centrifuge bottles on ice for 5 minutes, add 1.5 ml 5 M KAc (PH 5.2) and continue to freeze for 30 minutes. Then 10000 g centrifuge 15 min, discarding the pellet.

10. Extracted the supernatant with an equal volume of saturated phenol and chloroform∶isoamyl-alcohol (24∶1) in the centrifugation of 10000 g 20 min for twice.

11. Added an equal volume of isopropyl alcohol (about 10 ml) to the upper clear aqueous phase. Then put the centrifuge bottles in the −20°C for 1 hour or overnight.

12. Centrifuged the aqueous phase at 10000 g for 20 min. The cpDNA pellet was washed repeatedly with ethanol (70%, 96%), air-dried, and re-dissolved in 50 µl TE buffer.

13. Treated the cpDNA sample with 2 µl RNAse and visualize the DNA band on a 0.8% agarose gel.

B. high salt method [13]

Reagents

Cold isolation buffer: 1.25 M NaCl, 50 mM Tris-HCl (pH 8.0), 5 mM EDTA, 0.1% BSA (w/v), 0.1% β-mercaptoethanol (v/v).

Chloroplast isolation

All the following steps were carried out at 0°C if not otherwise stated.

1. Prior to extraction, about 20 g (fresh weight) leaves were collected and kept in dark for 48 to 72 hours at 4°C in order to decrease starch level stored in the leaves.

2. The leaves were cut into pieces (∼1 cm) and homogenized in 100 ml ice-cold Cold isolation buffer for 30 seconds. Filter the homogenate into centrifuge bottles using two layers of Miracloth (Merck) with softly squeezing the cloth.

3. Centrifuged the homogenate (3000 g, 10 min).

4. Resuspended the chloroplast pellet in 30 ml cold isolation buffer, and repellet the chloroplasts (3000 g, 10 min).

5. Resuspended the final chloroplast pellet in 10 ml cold isolation buffer.

Chloroplast DNA isolation

6. Added 1/10 volume of 10% CTAB to lyse the chloroplasts. Incubate at 55°C for 1 to 2 hours.

7. Extracted the supernatant with an equal volume of saturated phenol and chloroform∶isoamyl-alcohol (24∶1) in the centrifugation of 10000 g 20 min for twice.

8. Added an equal volume of isopropyl alcohol (about 10 ml) to the upper clear aqueous phase. Then put the centrifuge bottles in the −20°C for 1 hour or overnight.

9. Centrifuged the aqueous phase at 10000 g for 20 min. The cpDNA pellet is washed repeatedly with ethanol (70%, 96%), air-dried, and re-dissolved in 50 µl TE buffer.

10. Treated the cpDNA sample with 2 µl RNAse and visualize the DNA band on a 0.8% agarose gel.

C. sucrose gradient centrifugation [9]

Reagents

Isolation buffer: 0.35 M sorbitol, 50 mM Tris–HCl (pH 8.0), 5 mM EDTA, 0.1% BSA, 0.1% β-mercaptoethanol (v/v);

wash buffer: 0.35 M sorbitol, 50 mM Tris–HCl (pH 8.0), 25 mM EDTA;

Chloroplast isolation

1. Prior to extraction, about 20 g (fresh weight) leaves were collected and kept in dark for 48 to 72 hours at 4°C in order to decrease starch level stored in the leaves.

2. The leaves were cut into pieces (∼1 cm) and homogenized in 400 ml ice-cold isolation buffer for 30 seconds. Filter the homogenate into centrifuge bottles using two layers of Miracloth (Merck) with softly squeezing the cloth.

3. Centrifuged the homogenate (1000 g, 20 min).

4. Resuspended pellet in 7 ml of ice-cold wash buffer using a soft paintbrush.

5. Gently loaded the resuspended pellet onto a step gradient consisting of 18 ml of 52% sucrose, overlayered with 7 ml of 30% sucrose.

6. Centrifuged the step gradients at 25,000 rpm for 60 min at 4°C in a swinging bucket rotor.

7. Removed the chloroplast band from the 30–52% interface using a wide-bore pipette, dilute with 40 ml wash buffer, and centrifuge at 1500 g for 15 min at 4°C.

8. Resuspended the chloroplast pellet with 2 ml wash buffer.

Chloroplast DNA isolation

9. Chloroplast DNA isolation followed steps 6–10 in high salt method.

D. DNAse I treatment [9]

In the DNAse I treatment method, steps were the same with sucrose gradient centrifugation method except the step 9 which was treated with DNAse I. That is, the step 9 in sucrose gradient centrifugation method was substituted with: add 20 µl DNAse I (10 mg/ml) and 250 µl 200 mM MgCl_2_ to chloroplast solution buffer, incubate at 37°C for 60 min. Then add 1 ml 0.5 M EDTA to terminate the reaction.

### Chloroplast genome sequencing and data analysis

After the cpDNA isolation with modified high salt method, approximately 5–10 µg of DNA was sheared, followed by adapter ligation and library amplification, subjecting to Illumina Sample Preparation Instructions. The fragmented cpDNAs were sequenced at both single-read using the Illumina Genome Analyzer IIx platform at the in-house facility at The Germplasm Bank of Wild Species in Southwestern China. The obtained paired-end reads (2×100 bp read lengths) were assembled to the reference genome sequence to roughly estimate the genome coverage and cpDNA purity (the reads aligned to the reference genome sequence were served as cpDNA sequence) using the software program Geneious version 4.7 [20]. The reference chloroplast genome sequence of *O. nivara* (NC_005973) and *P. persica* (NC_014697) were downloaded from GenBank.

## References

[1] Sugiura M (1995). The chloroplast genome.. Essays Biochem.

[2] Sugiura M (1992). The chloroplast genome.. Plant Mol Biol.

[3] Moore MJ, Bell CD, Soltis PS, Soltis DE (2007). Using plastid genome-scale data to resolve enigmatic relationships among basal angiosperms.. Proc Natl Acad Sci USA.

[4] Jansen RK, Cai ZQ, Raubeson LA, Daniell H, dePamphilis CW (2007). Analysis of 81 genes from 64 plastid genomes resolves relationships in angiosperms and identifies genome-scale evolutionary patterns.. Proc Natl Acad Sci USA.

[5] Moore MJ, Soltis PS, Bell CD, Burleigh JG, Soltis DE (2010). Phylogenetic analysis of 83 plastid genes further resolves the early diversification of eudicots.. Proc Natl Acad Sci USA.

[6] Moore M, Dhingra A, Soltis P, Shaw R, Farmerie W (2006). Rapid and accurate pyrosequencing of angiosperm plastid genomes.. BMC Plant Biol.

[7] Shaffer C (2007). Next-generation sequencing outpaces expectations.. Nat Biotechnol.

[8] Heinze Berthold (2007). A database of PCR primers for the chloroplast genomes of higher plants.. Plant Methods.

[9] Jansen RK, Raubeson LA, Boore JL, dePamphilis CW, Chumley TW (2005). Methods for obtaining and analyzing whole chloroplast genome sequences.. Methods Enzymol.

[10] Diekmann K, Hodkinson TR, Fricke E, Barth S (2008). An optimized chloroplast DNA extraction protocol for grasses (Poaceae) proves suitable for whole plastid genome sequencing and SNP detection.. PLoS ONE.

[11] Cronn R, Liston A, Parks M, Gernandt DS, Shen RK (2008). Multiplex sequencing of plant chloroplast genomes using Solexa sequencing-by- synthesis technology.. Nucleic Acids Res.

[12] Kolodner R, Tewari KK (1979). Inverted repeats in chloroplast DNA from higher plants.. Proc Natl Acad Sci USA.

[13] Bookjans G, Stummann BM, Henningsen KW (1984). Preparation of chloroplast DNA from pea plastids isolated in a medium of high ionic-strength.. Anal Biochem.

[14] Lang BF, Burger G (2007). Purification of mitochondrial and plastid DNA.. Nature Protoc.

[15] Palmer JD (1986). Isolation and structural analysis of chloroplast DNA.. Methods Enzymol.

[16] Parks M, Cronn R, Liston A (2009). Increasing phylogentic resolution at low taxonomc levels using massively parallel sequencing of chloroplast genomes.. BMC Biol.

[17] Nock CJ, Waters DLE, Edwards MA, Bowen SG, Rice N (2010). Chloroplast genome sequences from total DNA for plant identification.. Plant Biotechnol J.

[18] Tangphatsornruang S, Sangsrakru D, Chanprasert J, Uthaipaisanwong P, Yoocha T (2010). The chloroplast genome sequence of mungbean (Vigna radiata) determined by high-throughput pyrosequencing: structural organization and phylogenetic relationships.. DNA Res.

[19] Atherton RA, McComish BJ, Shepherd LD, Berry LA, Albert NW (2010). Whole genome sequencing of enriched chloroplast DNA using the Illumina GAII platform.. Plant Methods.

[20] Drummond AJ, Ashton B, Cheung M, Heled J, Kearse M (2009). http://www.geneious.com.

